# Cost-effectiveness of Pembrolizumab Plus Axitinib Vs Nivolumab Plus Ipilimumab as First-Line Treatment of Advanced Renal Cell Carcinoma in the US

**DOI:** 10.1001/jamanetworkopen.2020.16144

**Published:** 2020-10-14

**Authors:** Tina R. Watson, Xin Gao, Kerry L. Reynolds, Chung Yin Kong

**Affiliations:** 1 Institute for Technology Assessment, Massachusetts General Hospital, Boston; 2 Massachusetts General Hospital Cancer Center, Boston; 3 Harvard Medical School, Boston, Massachusetts

## Abstract

**Importance:**

Checkpoint inhibitor combination therapy represents a major advance in the first-line treatment of advanced renal cell carcinoma. Pembrolizumab-axitinib and nivolumab-ipilimumab have become standard of care options after demonstrating clinical efficacy against sunitinib in separate phase 3 trials. The cost-effectiveness of these regimens is unknown.

**Objective:**

To evaluate the cost-effectiveness of pembrolizumab-axitinib and nivolumab- ipilimumab in the first-line treatment of advanced renal cell carcinoma.

**Design, Setting, and Participants:**

For this economic evaluation, a primary microsimulation model was developed and run between August and December 2019. Separate analyses were conducted for an intermediate- and poor-risk patient population (base case) and a favorable-risk population (exploratory analysis) because prognosis is known to differ between risk groups; 100 000 patients with advanced renal cell carcinoma were simulated in each treatment arm. Survival, treatment regimens, and other relevant conditions were based on data from the phase 3 KEYNOTE-426 and CheckMate214 clinical trials. The study perspective was the US health care sector.

**Main Outcomes and Measures:**

An incremental cost-effectiveness ratio was calculated for each of the 2 analyses and compared with a willingness-to-pay threshold of $100 000 per quality-adjusted life-year (QALY).

**Results:**

Pembrolizumab-axitinib was estimated to add 0.60 QALYs compared with nivolumab-ipilimumab in the base case analysis (3.66 vs 3.05 QALYs) and 0.25 QALYs compared with nivolumab-ipilimumab in the exploratory analysis (4.55 vs 4.30 QALYs), and was more costly (base case analysis: $562 927 vs $458 961; exploratory analysis: $589 035 vs $470 403). The incremental cost-effectiveness ratio was $172 532 per QALY in the base case analysis and $468 682 per QALY in the exploratory analysis. One-way sensitivity analyses revealed that the base case model was most sensitive to first-line drug prices (incremental cost-effectiveness ratio at upper limit of nivolumab price and lower limits of axitinib and pembrolizumab prices: $89 983, $102 287, and $114 943 per QALY, respectively). The exploratory analysis model was most sensitive to overall survival rates (incremental cost-effectiveness ratio at lower limit of pembrolizumab-axitinib rate and upper limit of nivolumab-ipilimumab rate: $278 644 and $285 684 per QALY, respectively).

**Conclusions and Relevance:**

The findings suggest that pembrolizumab-axitinib treatment is associated with greater QALYs compared with nivolumab/ipilimumab treatment in patients with advanced renal cell carcinoma but may not be cost-effective. Price reductions may make the cost of pembrolizumab-axitinib proportional to its clinical value and less financially burdensome to the US health care system.

## Key Points

**Question:**

Is pembrolizumab-axitinib cost-effective as first-line treatment of advanced renal cell carcinoma compared with nivolumab-ipilimumab, the other preferred first-line regimen?

**Findings:**

In this economic evaluation using a microsimulation model, pembrolizumab-axitinib provided incremental benefit over nivolumab-ipilimumab by a measure of quality-adjusted life-years but was not cost-effective at a willingness-to-pay threshold of $100 000 per quality-adjusted life-year.

**Meaning:**

The findings suggest that pembrolizumab-axitinib may be a valuable therapy for advanced renal cell carcinoma but may become more cost-effective with price reductions.

## Teaser

This economic evaluation compares the cost-effectiveness of pembrolizumab-axitinib vs nivolumab-ipilimumab for treatment of advanced renal cell carcinoma (ARCC) in the US.

## Introduction

Between 2018 and 2019, the US Food and Drug Administration (FDA) approved 3 first-line immune checkpoint inhibitor combination therapies for advanced renal cell carcinoma (RCC).^1,2,3^ The dual checkpoint inhibitor combination of nivolumab-ipilimumab was the first to show efficacy, followed by pembrolizumab and avelumab each in combination with axitinib, a vascular endothelial growth factor receptor (VEGFR) inhibitor.^1,2,3^ Although pembrolizumab-axitinib demonstrated efficacy by overall survival (OS), avelumab plus axitinib has not. Sunitinib, another antiangiogenic VEGFR inhibitor considered a standard of care in the decade before 2018, was the control in each of the phase 3 clinical trials leading to the approvals.^4,5,6^ As is reflected in the National Comprehensive Cancer Network treatment guidelines released in August 2019, nivolumab-ipilimumab and pembrolizumab-axitinib have replaced sunitinib as favored first-line therapies for advanced RCC, but there is no consensus on which is preferred.^5,7,8,9^

Advanced RCC is characterized by substantial variability in prognosis. Historically, patients have been classified as having favorable, intermediate, or poor risk based on several established clinical and laboratory risk factors.^10^ In the era of VEGFR therapy, the International Metastatic Renal Cell Carcinoma Database Consortium (IMDC) model has demonstrated an approximately 6-fold difference in OS across the range of risk.^10^ Nivolumab-ipilimumab was approved for IMDC intermediate- and poor-risk patients only because trial end points initially did not show compelling evidence of benefit of nivolumab-ipilimumab over sunitinib in the IMDC favorable-risk population. Of note, the IMDC criteria were developed to assess outcomes of antiangiogenic VEGFR monotherapy, indicating that favorable-risk patients are those who, by definition, have particularly VEGFR-responsive or otherwise indolent disease.^10^ The rationale for reserving nivolumab-ipilimumab for higher-risk patients only is unknown. Extended follow-up data from the CheckMate214 clinical trial,^11^ published in August 2019, report a complete response rate of 8% with nivolumab-ipilimumab vs 4% with sunitinib among favorable-risk patients; 90% of responses to nivolumab-ipilimumab were durable at a median follow-up of 32.4 months. Superior duration and depth of response are characteristic of immunotherapy as opposed to VEGFR therapy and have been associated with more traditional measures of clinical value, such as OS and quality of life.^8,12^

The enthusiasm surrounding the enhanced efficacy associated with checkpoint inhibitor therapies must be balanced against cost considerations and the realities of metastatic disease outcomes. When antiangiogenic VEGFR monotherapy was the standard of care, the 5-year survival rate for patients with advanced RCC was 12%.^13,14^ The extent to which nivolumab-ipilimumab and pembrolizumab-axitinib will improve outcomes remains to be determined. It is also uncertain which treatments provide additional clinical value cost-effectively compared with alternative treatments. The relevance of sunitinib as a comparator in these analyses was weakened by the clinical preference for newer treatments in real-world scenarios.

The cost-effectiveness of first-line RCC treatment with pembrolizumab-axitinib has not, to our knowledge, been evaluated in the US. Cost-effectiveness estimates for any first-line treatment of favorable-risk RCC specifically are also unavailable. Because of the drastic prognostic differences among patients with advanced RCC, cost-effectiveness analyses should examine IMDC risk groups separately to generate results most relevant to clinical practice. The primary objective of our study was to estimate the cost-effectiveness of pembrolizumab-axitinib compared with nivolumab-ipilimumab for intermediate- and poor-risk patients. In an additional exploratory analysis, we evaluated the cost-effectiveness of the 2 checkpoint inhibitor combinations for favorable-risk patients also if nivolumab-ipilimumab were to become a first-line option.

## Methods

### Simulation Model

This economic evaluation used a decision analytic microsimulation model to estimate the cost and effectiveness associated with nivolumab-ipilimumab and pembrolizumab-axitinib as first-line treatments for stage IV RCC from the US health care sector perspective. The analyses were conducted from August and December 2019.This study was deemed exempt from institutional review board approval by Massachusetts General Hospital. This study followed the Consolidated Health Economic Evaluation Reporting Standards (CHEERS) reporting guideline.^15^

**Figure 1.**
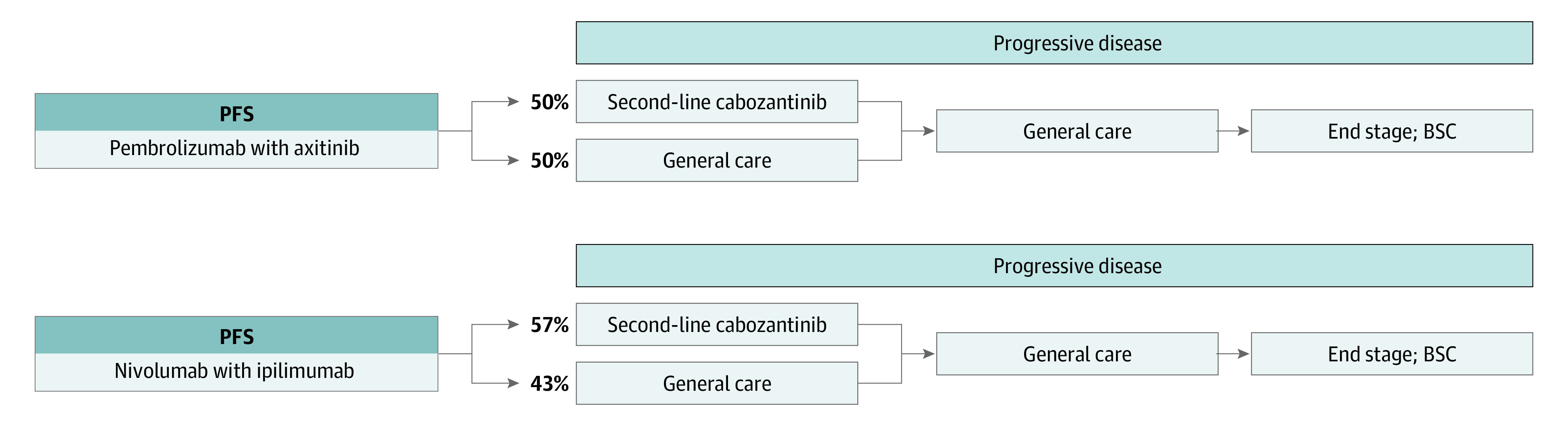
Model Schematic for the Base Case and Exploratory Analyses Separate models were built for the base case and exploratory analyses. Each incorporated the progression-free survival (PFS) and overall survival data specific to the intermediate- and poor-risk and favorable-risk patient populations, respectively.

Patients were simulated through 3 mutually exclusive health states: progression-free survival (PFS), progressive disease, and death. All began in PFS with advanced disease and at any time could die or move to the progressive disease state, in which a proportion of patients received second-line treatment (Figure 1). The 2 first-line treatments evaluated were (1) nivolumab-ipilimumab for four 3-week cycles followed by nivolumab monotherapy, based on the CheckMate214 trial,^4^ and (2) pembrolizumab-axitinib for a maximum of thirty-five 3-week cycles followed by axitinib monotherapy, following the KEYNOTE-426 trial.^6^ The basis of this cross-trial comparison was the demographic and clinical similarity between the 2 patient populations as well as closely correlating survival data between the sunitinib treatment arms used as the comparator in each trial (eTable 1 in the Supplement).

In the base case analysis, we considered only IMDC intermediate- and poor-risk patients. We conserved the same model structure in an exploratory analysis of IMDC favorable-risk patients with RCC. The CheckMate214 and KEYNOTE-426 trials reported significant differences in PFS and OS between the 2 patient subgroups (intermediate and poor risk vs favorable risk) receiving the same treatment.^6,10,11,16^ We thus performed separate analyses to capture the survival trajectories of each subgroup. Treatment regimens, adverse event rates, and proportions of patients receiving second-line therapy were derived from clinical trial data.^4,6,11,18,19,20,21,22,23,25,26,27,28,29^ First-line drug doses followed FDA-approved prescribing information.^1,2^ Table 1 summarizes all model inputs.

**Table 1.** Model Inputs

Variable	Input value	Source
**Cost inputs, price per mg**		
Axitinib, $	53.01^a^	Micromedex Red Book^17^
Cabozantinib price per tablet, $	674.59^a^	Micromedex Red Book^17^
Ipilimumab, $	156.47	CMS October 2019 ASP Drug Pricing Files^18^
Nivolumab price per mg, $	28.41	CMS October 2019 ASP Drug Pricing Files^18^
Pembrolizumab price per mg, $	50.47	CMS October 2019 ASP Drug Pricing Files^18^
Immunotherapy administration cost, $	146.20	CMS Physician Fee Schedule^19^ (*Current Procedural Terminology* code 96413)
General treatment and monitoring, $	2095.00	Benedict et al,^20^ 2011
General treatment in BSC, $	11 122.86	Henk et al,^21^ 2013; Sheehan et a1,^22^ 2019
Death-related costs, $	10 329.97	Perrin et al,^23^ 2014
Pembro-axi AE weighted mean cost per patient, $	3393.27	Agency for Healthcare Research and Quality^24^; Rini et al,^6^ 2019
Nivo-ipi AE weighted mean cost per patient, $	2862.07	Agency for Healthcare Research and Quality^24^; Motzer et al,^4^ 2018
**Utilities**^b^		
Pembro-axi PFS utility	0.77	Details in eMethods of the Supplement
Nivo-ipi PFS utility	0.82	Motzer et al,^4^ 2018; Wan et al,^25^ 2019
PD utility	0.66	De Groot et al,^26^ 2018
**Other**		
Average patient weight, kg	71.40	McCrea et al,^27^ 2018; Portier et al,^28^ 2007
Second-line therapy proportion		
Pembro-axi	0.50	Rini et al,^6^ 2019
Nivo-ipi	0.57	Motzer et al,^11^ 2019

Abbreviations: AE, adverse event; ASP, average sales price; BSC, best supportive care; CMS, Centers for Medicare & Medicaid Services; nivo-ipi, nivolumab-ipilimumab; pembro-axi, pembrolizumab-axitinib; PD, progressive disease; PFS, progression-free survival.

^a^ Multiplied by 0.86.^23^

^b^ Health state utility values are a universal component of cost effectiveness analyses that are understood to mean the desirability of the given health state, with 0 indicating death and 1 indicating perfect health. Thus, the input values have no units.

The model outputs were the total cost of treatment per patient, the effectiveness of treatment as measured by quality-adjusted life-years (QALYs), and the incremental cost-effectiveness ratio (ICER) between the 2 strategies. ICERs were compared with a willingness-to-pay (WTP) threshold of $100 000 per QALY.^30^

### Survival and Health State Utilities

In the base case analysis, PFS and OS rates in the nivolumab-ipilimumab treatment arm were derived from Kaplan-Meier survival curves from the extended follow-up of CheckMate214.^11^ After month 30, significant censoring compromised the reliability of the trial data; exponential functions provided the best fit to the PFS and OS curves and were used thereafter to project survival. After month 60, OS was projected using observed mortality rates from Surveillance, Epidemiology, and End Results data on adult patients with advanced RCC.^31,32^ Separate KM curves by IMDC risk category were not published in KEYNOTE-426. Therefore, median PFS and 12-month OS rates by IMDC risk were used to project PFS and OS in the pembrolizumab-axitinib treatment arm.^6^ As in the nivolumab-ipilimumab group, SEER mortality rates were applied after month 60 to project OS.^31,32^ In the exploratory analysis, the same methods were applied using PFS and OS Kaplan-Meier curves specific to favorable-risk patients receiving nivolumab-ipilimumab the corresponding median PFS and 12-month OS rate for those receiving pembrolizumab-axitinib .^6,11^ (eFigures 1 and 2 in the Supplement).

The nivolumab-ipilimumab PFS utility was derived from the literature and based on quality of life assessments in the CheckMate214 trial using the Functional Assessment of Cancer Therapy-Kidney Symptom Index.^4,25^ Because quality of life data from KEYNOTE-426 were not yet available at the time of this analysis, we estimated a pembrolizumab-axitinib PFS utility on the basis of expert opinion.^25,33^ More detail can be found in the eMethods in the Supplement. Treatment-specific utilities were applied regardless of IMDC risk. A previously published utility for RCC progressive disease was applied after patients left the PFS health state in each treatment arm.^26^

### Cost Estimates

We considered the costs of first- and second-line drug acquisition and administration, general treatment associated with PFS including follow-up and monitoring, best supportive care, adverse event management, and death from RCC.^4,6,17,18,19,20,21,22,23,24,29^ Drug costs were sourced from the Centers for Medicare & Medicaid Services October 2019 Average Sales Price Drug Pricing Files and the IBM Micromedex Red Book.^17,18^ For drugs dependent on body weight, we used a mean body weight from the literature for patients with advanced RCC in the US.^27,28^ Immunotherapy administration costs were taken from the CMS Physician Fee Schedule.^19^ In calculating adverse event management costs, we considered only events of grade 3 or higher and occurring in 1% or more of the clinical trial populations.^6,11^ Each adverse event was matched to an *International Classification of Diseases, Ninth Revision* code. Median costs per patient corresponding to each diagnosis code were sourced from the Healthcare Cost and Utilization Project, a network of health care databases sponsored by the Agency for Healthcare Research and Quality.^24^ To calculate the weighted mean cost per patient, we multiplied the proportion of patients in the clinical trials who experienced each adverse event by the corresponding cost of management and then combined all costs to get the aggregate value. Costs of general treatment, best supportive care, and death from RCC were sourced from the literature.^20,21,22,23^ Additional information on costs are given in the eMethods in the Supplement. All costs were adjusted to 2020 US dollars using the Centers for Medicare & Medicaid Services Personal Healthcare Price Index.^34,35^

### Statistical Analysis

TreeAge Pro statistical software, version 2019 R1 (TreeAge Software, LLC) was used to perform the analysis. The cycle length was 1 month. Cost and survival estimates were discounted at an annual rate of 3%. One-way deterministic sensitivity analyses were performed in TreeAge. Relevant variables were tested at the upper and lower limits of plausible ranges (eTable 2 in the Supplement).

Sensitivity analyses were conducted separately for the base case and exploratory analyses. To determine the effect of variation in multiple variables at once, we performed a probabilistic sensitivity analysis with 1000 iterations assessing the variable ranges listed in eTable 2 in the Supplement. Additional information on the probabilistic sensitivity analysis is given in eFigure 3 in the Supplement. Uncertainty in simulation modeling results is most appropriately captured by sensitivity analyses rather than 95% CIs because the 95% CIs could be arbitrarily large or small depending on the number of simulated patients.

## Results

### Incremental Cost-effectiveness Ratios

In the base case, first-line treatment with nivolumab-ipilimumab resulted in a mean cancer-attributable cost of $458 961 and mean survival of 3.05 QALYs per patient. Treatment with pembrolizumab-axitinib resulted in a mean cost of $562 927 and mean survival of 3.66 QALYs. Pembrolizumab-axitinib cost an additional $103 966 and conferred an additional 0.60 QALYs, yielding an ICER of $172 532 per QALY (Table 2).

**Table 2.** Summary of Simulation Results

Analysis	Cost, $	QALYs	Incremental		ICER (incremental cost/QALY), $
			Cost, $	QALYs	
Base case					
Nivo-ipi	458 961	3.05	NA	NA	NA
Pembro-axi	562 927	3.66	103 966	0.60	172 532
Exploratory					
Nivo-ipi	470 403	4.30	NA	NA	NA
Pembro-axi	589 035	4.55	118 632	0.25	468 682

Abbreviations: ICER, incremental cost-effectiveness ratio; nivo-ipi, nivolumab-ipilimumab; NA, not applicable; pembro-axi, pembrolizumab-axitinib; QALYs, quality-adjusted life-years.

In the exploratory analysis, treating patients with nivolumab-ipilimumab in the first line was associated with a mean cost of $470 403 and mean survival of 4.30 QALYs. The pembrolizumab-axitinib treatment strategy cost $589 035 on average and yielded a mean survival of 4.55 QALYs. The incremental cost was $118 632 and incremental benefit was 0.25 QALYs. The ICER was estimated to be $468 682 per QALY (Table 2).

In the base case, a 32% reduction in the price of pembrolizumab or a 27% reduction in the price of axitinib allowed pembrolizumab-axitinib to become cost-effective, as did a 15% reduction in the price of both drugs. In the exploratory analysis, the regimen became cost-effective with either a 68% or 58% reduction in the prices of pembrolizumab and axitinib, respectively, as well as a 35% reduction in the price of both drugs.

### Sensitivity Analysis

**Figure 2.**
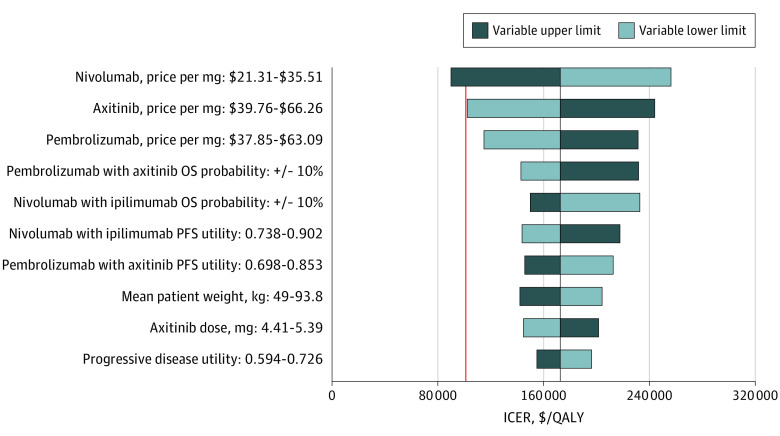
Deterministic Sensitivity Analysis for the Base Case Analysis The vertical red line represents the $100 000 per quality-adjusted life-year (QALY) willingness-to-pay threshold we used in our analysis. The vertical black line represents the primary result of $172 532 per QALY as the incremental cost-effectiveness ratio (ICER) in the base case. OS indicates overall survival; PFS, progression-free survival.

In 1-way sensitivity analyses conducted for the base case analysis, pembrolizumab-axitinib was cost-effective assuming a WTP threshold of $100 000 per QALY only at the upper limit of nivolumab price, with an estimated ICER of $89 983 per QALY. Large decreases in the ICER also occurred at the lower limits of axitinib and pembrolizumab price, although each remained just above the WTP threshold ($102 287 and $114 943 per QALY, respectively). Mean patient weight at the variable upper limit and the OS probability associated with pembrolizumab-axitinib at the variable lower limit also brought the ICER close to the WTP threshold, at $142 126 and $142 861 per QALY, respectively. The 10 variables with the greatest associations with the ICER are shown in Figure 2.

**Figure 3.**
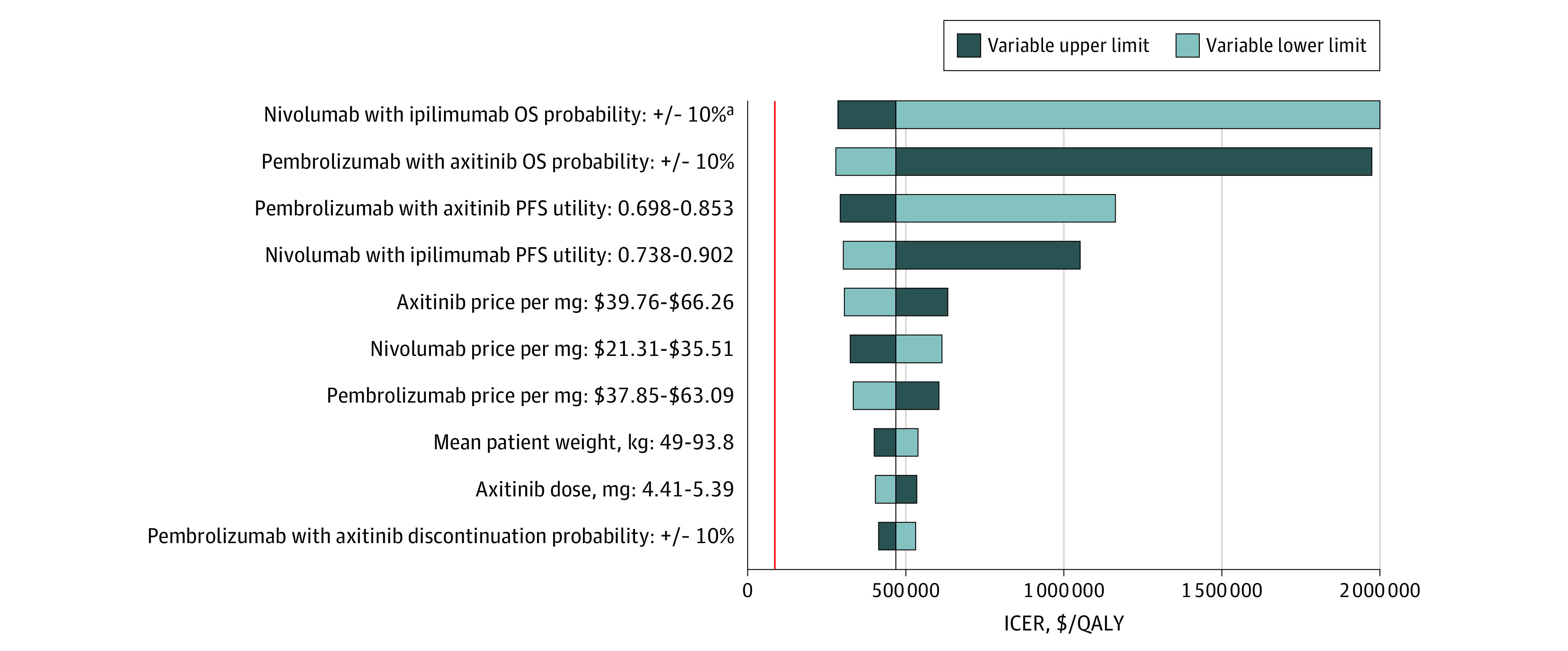
Deterministic Sensitivity Analysis for the Exploratory Analysis The vertical red line represents the $100 000 per quality-adjusted life-year (QALY) willingness-to-pay threshold. The vertical black line represents the primary result of $468 682 per QALY as the incremental cost-effectiveness ratio (ICER) in the exploratory analysis. OS indicates overall survival; PFS, progression-free survival. ^a^The ICER at the lower limit of the nivolumab-ipilimumab OS probability was estimated to be $3 835 509 per QALY.

In the exploratory analysis, pembrolizumab-axitinib was not cost-effective at any of the tested variable upper or lower limits. The ICER was closest to the WTP threshold at the lower limit of the pembrolizumab-axitinib OS probability and the upper limit of the nivolumab-ipilimumab OS probability ($278 644 and $285 684 per QALY, respectively). As shown in Figure 3, the 10 variables that were most associated with the ICER were the same as those in the base case analysis excluding the replacement of the progressive disease utility variable with the probability of discontinuing pembrolizumab-axitinib.

The PSA of the base case revealed that pembrolizumab-axitinib was cost-effective in 9.7% of the 1000 iterations. Nivolumab-ipilimumab was dominated in 1 iteration. Treatment with pembrolizumab-axitinib had a 50% probability of being cost-effective at a WTP threshold of approximately $176 000 per QALY (eFigure 3A in the Supplement). In the exploratory population, pembrolizumab-axitinib was not cost-effective in any of the 1000 iterations and dominated in 4% of iterations. At a WTP threshold of approximately $482 000 per QALY, treatment with pembrolizumab-axitinib had a 50% probability of being cost-effective (eFigure 3B in the Supplement).

## Discussion

On the basis of a primary microsimulation model, we estimated that first-line advanced RCC treatment with pembrolizumab-axitinib regardless of IMDC risk was more expensive, on average, than treatment with nivolumab-ipilimumab and was associated with a greater survival benefit as measured by QALYs. In separate analyses of an intermediate- and poor-risk simulated patient population in the base case and a favorable risk population in the exploratory analysis, we estimated that pembrolizumab-axitinib was not cost-effective compared with nivolumab-ipilimumab when assuming a WTP threshold of $100 000 per QALY. This study is, to our knowledge, the first cost-effectiveness analysis to compare RCC treatment with pembrolizumab-axitinib in the US and the first to compare the 2 RCC checkpoint inhibitor combinations, which have recently become the preferred first-line treatment options.^7,36^ In addition, to our knowledge, this study is also the first to estimate the cost-effectiveness of first-line checkpoint inhibitor therapy in favorable-risk RCC patients specifically, who, by definition, respond well to antiangiogenic drugs but for whom optimal treatment remains debatable in the era of immunotherapy.^10^

The base case model was most sensitive to the price per milligram of nivolumab, axitinib, and pembrolizumab. After drug prices, OS probabilities had the greatest effect on the model results, underscoring the need for longer-term survival data to validate our results. The clearest realistic means of rendering the cost of pembrolizumab-axitinib proportionate to its clinical value, according to our model, would be to lower the price of pembrolizumab or axitinib. We found that either a 32% reduction in the price per milligram of pembrolizumab or a 27% reduction in the price of axitinib allowed pembrolizumab-axitinib to become cost-effective. Alternatively, a 15% reduction in the price per milligram of both pembrolizumab and axitinib was enough to make the regimen cost-effective. Further supporting the notion that pembrolizumab-axitinib is currently costly for its clinical value, trial data suggest its inferiority to nivolumab-ipilimumab, with a higher rate of grade 3 or higher treatment-related adverse events in an entire study population (62.9% vs 47%) and a lower rate of complete response in intermediate- and poor-risk populations (4.8% vs 11%).^6,11,37^ Additional studies comparing the cost-effectiveness of the 2 regimens with avelumab plus axitinib may be justified if further follow-up data demonstrate its improved performance over sunitinib.

In the exploratory analysis, pembrolizumab-axitinib was associated with a smaller incremental QALY gain than in the base case (0.25 vs 0.60 QALYs), largely explaining the comparatively high ICER. One-way sensitivity analyses confirmed that the results were most affected by variables determining QALY gain—probability of death and treatment-specific utility—rather than drug prices, as in the base case. In the favorable-risk population, the ICER was therefore not only higher but also associated with factors comparably more difficult to change through clinical or policy interventions. In addition, a 68% or 58% reduction in the prices per milligram of pembrolizumab and axitinib, respectively, was associated with an ICER below the WTP threshold, as was a 35% reduction in the price of both drugs. Our exploratory analysis, therefore, may not support for the use of any realistic interventions bringing pembrolizumab-axitinib closer to cost-effectiveness, but it may instead support a case for expanding the nivolumab-ipilimumab indication to favorable-risk patients, who constitute approximately one-fifth of the population with advanced RCC.^10^ The small survival discrepancy suggests that long-term prognosis associated with the 2 treatments may be similar among these patients, with less severe toxic effects from nivolumab-ipilimumab. Furthermore, the differences in efficacy outcomes between nivolumab-ipilimumab and sunitinib narrowed in extended follow-up data and may continue to do so.^11^ Our conclusions regarding the poor cost-effectiveness of pembrolizumab-axitinib may add an important dimension to the debate over treatment options for patients with favorable risk of RCC.

The outcomes of our base case analysis support the continued use of nivolumab-ipilimumab as a first-line treatment option for intermediate- and poor-risk patients. However, the survival benefit and higher likelihood of objective response associated with pembrolizumab-axitinib may be judged to be more important than its poor cost-effectiveness in individual treatment decisions. Previous studies have estimated nivolumab-ipilimumab to be cost-effective for intermediate- and poor-risk patients compared with sunitinib.^25,38,39^ Our choice not to model sunitinib represents an effort to reflect the recent shift in the RCC treatment paradigm away from sunitinib as the standard first-line therapy and toward checkpoint inhibitor combinations.

### Limitations

This study has limitations. First, quality of life data for patients receiving pembrolizumab-axitinib was unavailable; to evaluate an appropriate utility, we had to make certain assumptions based on quality of life–derived values for other first-line therapies for advanced RCC, incorporating toxic effects and response data (eMethods in the Supplement). We tested the robustness of our results by varying utility values in the sensitivity analysis and found that our conclusions did not change. Second, we modeled proportions of patients receiving second-line therapy based on clinical trial data, which may or may not reflect the real-world prevalence of second-line treatment. Second-line therapy data stratified by IMDC prognostic risk group were unavailable; thus, we used the same proportion for all patients receiving a given treatment (50% for pembrolizumab-axitinib and 57% for nivolumab-ipilimumab).^4,6^ In addition, our exploratory analysis should be interpreted with caution given the small favorable-risk patient population in each checkpoint inhibitor arm.^4,6^ More robust cost-effectiveness estimates are only possible with additional data as these therapies are further incorporated into practice and outcomes observed over time. Although there are limitations inherent in any simulation study comparing 2 separately randomized patient populations, it is uncertain whether a head-to-head trial comparing these 2 immunotherapy regimens will take place.

## Conclusions

In this economic evaluation comparing the 2 preferred first-line therapies for intermediate- and poor-risk patients with advanced RCC, we estimated that pembrolizumab-axitinib was associated with improved QALYs compared with nivolumab-ipilimumab but was not cost-effective. Our findings may support efforts to lower drug prices and render this treatment less of a financial burden on the US health care system. The findings of the exploratory analysis suggest that for favorable-risk patients, pembrolizumab-axitinib would be associated with benefit but at an additional cost. Further studies on the cost-effectiveness and long-term outcomes of nivolumab-ipilimumab for the treatment of patients with favorable risk for RCC are warranted.

## Notes

Supplement. **eTable 1.** Comparison of Patient Populations From KEYNOTE-426 and CheckMate214 **eTable 2.** Sensitivity Analysis Parameters **eFigure 1.** Progression-Free Survival Projections **eFigure 2.** Overall Survival Projections **eFigure 3.** Acceptability Curves for the Probabilistic Sensitivity Analysis **eMethods.** **eReferences.**
