# Supplementary material for: Cost-effectiveness of Pembrolizumab Plus Axitinib Vs Nivolumab Plus Ipilimumab as First-Line Treatment of Advanced Renal Cell Carcinoma in the US
Source: JAMA Netw Open. 2020 Oct 14;3(10):e2016144. doi: 10.1001/jamanetworkopen.2020.16144 (PMC7557509; doi:10.1001/jamanetworkopen.2020.16144)
Supplement: Supplement. — eTable 1. Comparison of Patient Populations From KEYNOTE-426 and CheckMate214 eTable 2. Sensitivity Analysis Parameters eFigure 1. Progression-Free Survival Projections eFigure 2. Overall Survival Projections eFigure 3. Acceptability Curves for the Probabilistic Sensitivity Analysis eMethods. eReferences. [file jamanetwopen-e2016144-s001.pdf]

Supplemental Online Content

Watson TR, Gao X, Reynolds KL, Kong CY. Cost-effectiveness of pembrolizumab plus axitinib versus nivolumab plus ipilimumab as first-line treatment of advanced renal cell carcinoma in the US. *JAMA Netw Open*. 2020;3(10):e2016144. doi:10.1001/jamanetworkopen.2020.16144

eTable 1. Comparison of Patient Populations from KEYNOTE-426 and CheckMate214

eTable 2. Sensitivity Analysis Parameters

eFigure 1. Progression-free survival projections

eFigure 2. Overall survival projections

eFigure 3. Acceptability curves for the probabilistic sensitivity analysis.

eMethods.

eReferences.

This supplemental material has been provided by the authors to give readers additional information about their work.

© 2020 Watson TR et al. *JAMA Network Open.*

eTable 1. Comparison of Patient Populations from KEYNOTE-426 and CheckMate214.

Characteristic	KEYNOTE-426[1]	CheckMate214[2,3]
Median age (range)	62 (30-89)	62 (26-85)
Male sex	71.3%	75%
Poor risk patients (in the base case population)	19.1%	21.4%
Patients with sarcomatoid features[a]	17.9%	14.2%
Previous radiotherapy	9.5%	11%
Previous nephrectomy	82.6%	82%
Median PFS on sunitinib (mos.)	11.1	9.7
12-mo OS rate on sunitinib	78.3%	78%
18-mo OS rate on sunitinib	72.1%	67.4%

PFS, progression-free survival; OS, overall survival

[a] Sarcomatoid histology in RCC is known to be associated with worse prognosis and greater metastatic potential, regardless of type of therapy.[4] Sarcomatoid prevalence is reported out of the total number of patients with known status, determined in the entire study population from KEYNOTE-426 but only in the intermediate or poor risk subgroup from CheckMate214.

© 2020 Watson TR et al. *JAMA Network Open.*

eTable 2. Sensitivity Analysis Parameters

	Model Input		Lower Bound	Upper Bound
**Costs[a]**				
Axitinib price per mg	$53.01		$39.76	$66.26
Cabozantinib price per tablet	$674.59		$505.94	$843.24
Ipilimumab price per mg	$156.47		$117.35	$195.59
Nivolumab price per mg	$28.41		$21.31	$35.51
Pembrolizumab price per mg	$50.47		$37.85	$63.09
General treatment/follow-up per cycle	$2,095.00		$1,571.25	$2,618.75
Best supportive care per cycle (last four cycles)	$11,122.86		$8,342.15	$13,903.58
Death cost (one-off)	$10,329.97		$7,747.48	$12,912.46
**Utilities**				
Pembro/Axi PFS	0.775		0.698	0.853
Nivo/Ipi PFS	0.82		0.738	0.902
Progressive disease	0.66		0.594	0.726
**Survival and Treatment Time**	**Base Case**	**Exploratory**		
Pembro/Axi progression probability	0.042	0.033	+/- 10%	
Nivo/Ipi progression probability	KM	KM	+/- 10%	
Pembro/Axi OS probability	0.011 <60 mos., then SEER <140 mos., then 0.03	0.006 <60 mos., then SEER <140 mos., then 0.03	+/- 10%	
Nivo/Ipi OS probability	KM <60 mos., then SEER <140 mos., then 0.03	KM <60 mos., then SEER <140 mos., then 0.03	+/- 10%	
Pembro/Axi treatment discontinuation probability	0.035 <96 mos., then 1	0.052 <96 mos., then 1	+/- 10%	
Nivo/Ipi treatment discontinuation probability	varying <96 mos., then 1	varying <96 mos., then 1	+/- 10%	
Cabozantinib treatment discontinuation probability	calibrated to match ToT from Choueiri, et al., 2015	calibrated to match ToT from Choueiri, et al., 2015	+/- 10%	
**Other**				
Axitinib dose, twice daily (mg)	4.9		4.41	5.39
Average patient weight (kg)	71.4		49.00	93.8
Pembro/Axi subsequent therapy proportion	0.5		0.45	0.55
Nivo/Ipi subsequent therapy proportion	0.57		0.513	0.627

Pembro/axi, pembrolizumab plus axitinib; nivo/ipi, nivolumab plus ipilimumab; PFS, progression-free survival; OS, overall survival; KM, Kaplan-Meier estimates; ToT, time on treatment

[a] Cost ranges indicate a 25% adjustment. All other ranges indicate a 10% adjustment.

© 2020 Watson TR et al. *JAMA Network Open.*

eFigure 1. Progression-free survival projections.

(a) Base case.

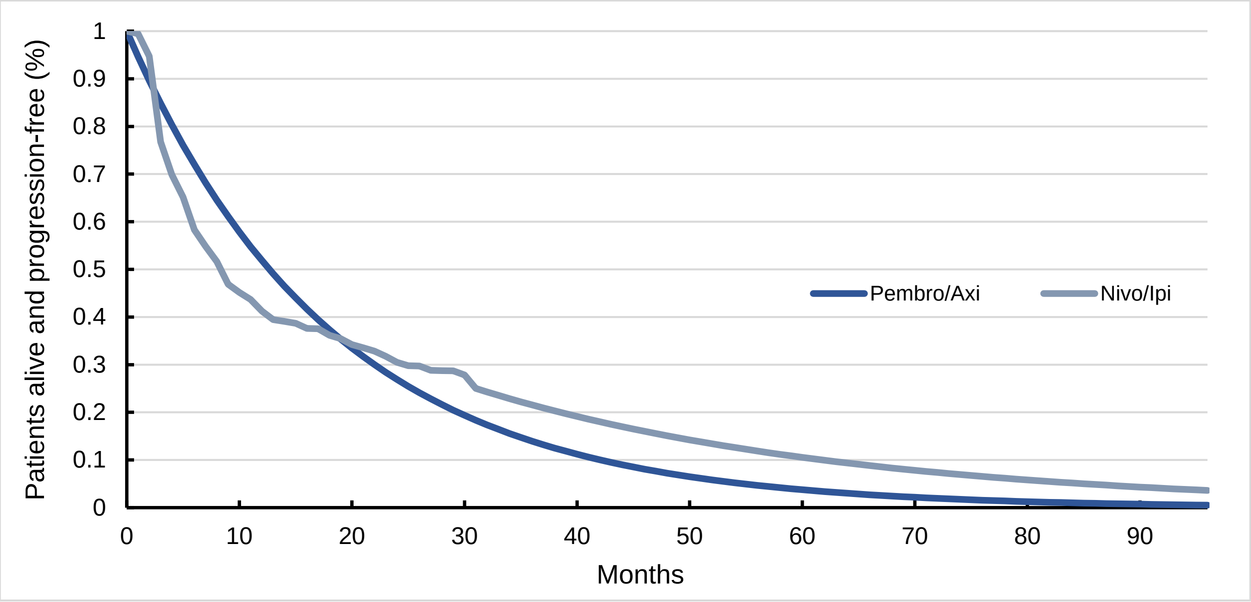

(b) Exploratory analysis.

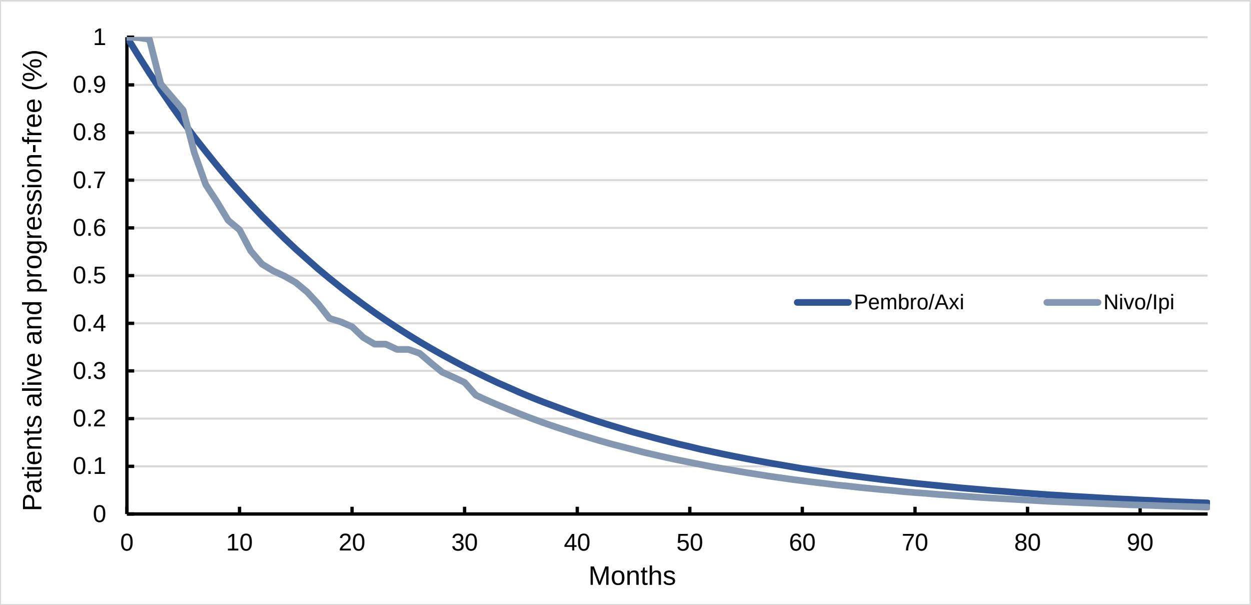

For the nivo/ipi arm in the base case and exploratory analysis, progression-free survival (PFS) rates were derived from Kaplan-Meier data from CheckMate214. The median duration of PFS in the model was matched to data from CheckMate214. For the pembro/axi arm in the base case, PFS was projected using an exponential function derived from the 12.67-month median reported in KEYNOTE-426 (a weighted average of the median PFS among intermediate and poor risk patients). For the exploratory analysis, PFS rates were based on the 17.7-month median time as reported in KEYNOTE-426.

© 2020 Watson TR et al. *JAMA Network Open.*

eFigure 2. Overall survival projections.

(a) Base case.

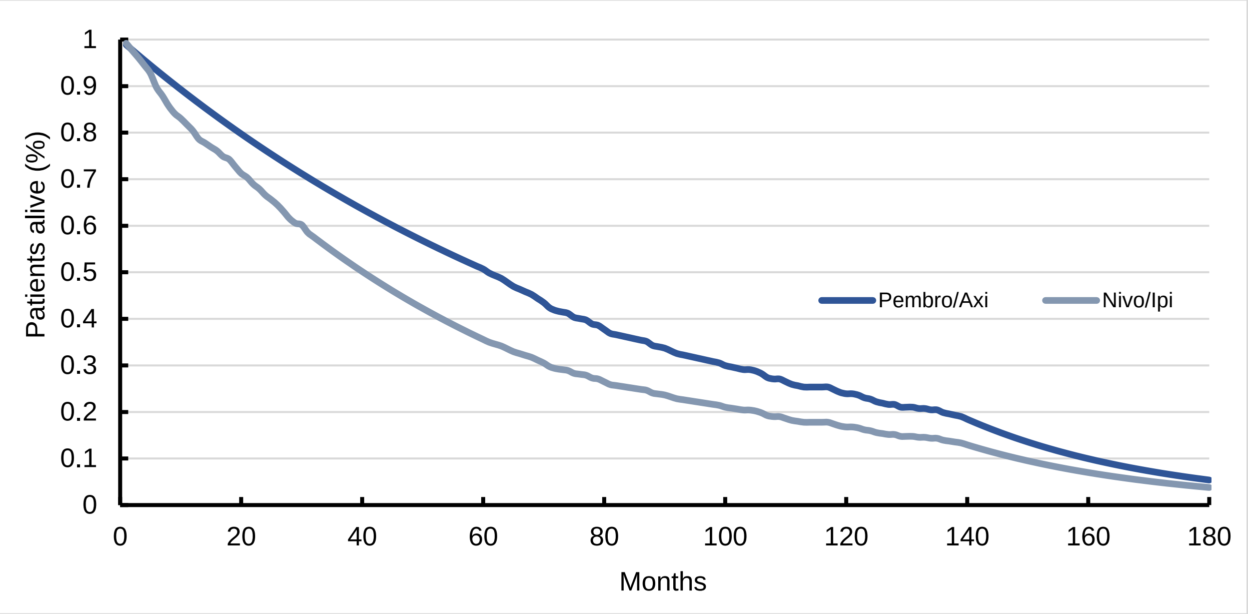

(b) Exploratory analysis.

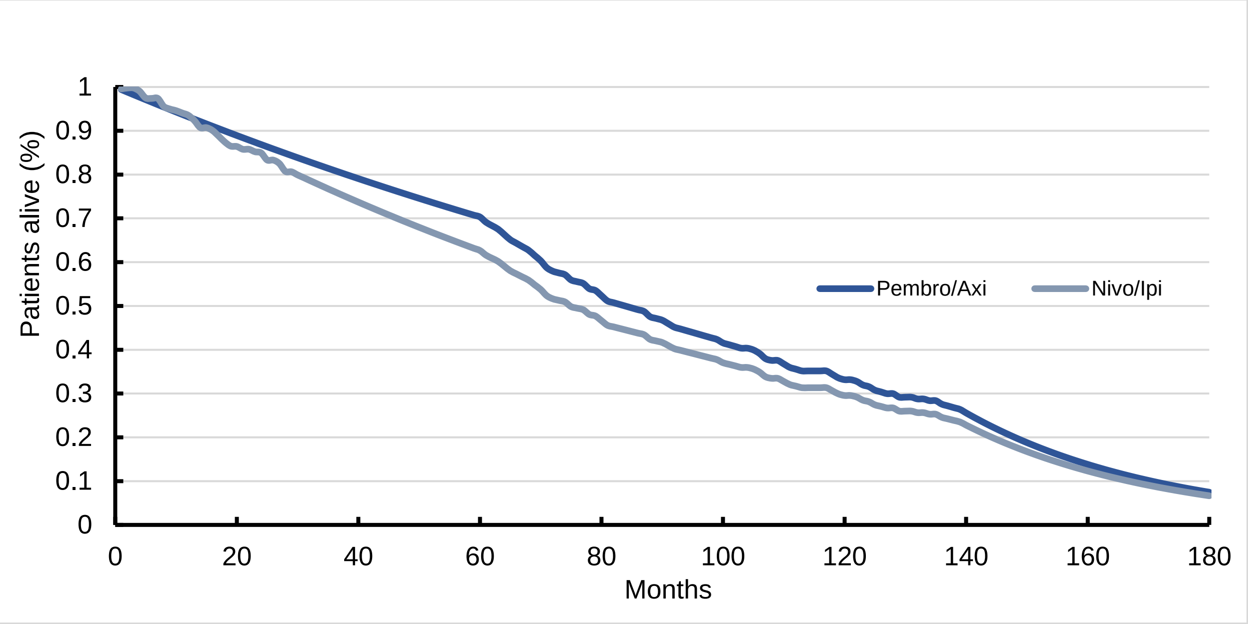

For the nivo/ipi arms, overall survival (OS) was projected using KM data, followed by exponential functions based on the KM curves, followed by SEER mortality rates. For the pembro/axi arms, OS projections were based on the OS rates provided in KEYNOTE-426 by IMDC risk group, followed by SEER mortality rates. After 140 months post-diagnosis, the sample size in SEER data was insufficiently large to generate reliable estimates; we therefore applied the last available conditional rate of death (3%) to all consecutive cycles.

© 2020 Watson TR et al. *JAMA Network Open.*

eFigure 3. Acceptability curves for probabilistic sensitivity analysis.

(a) Base case.

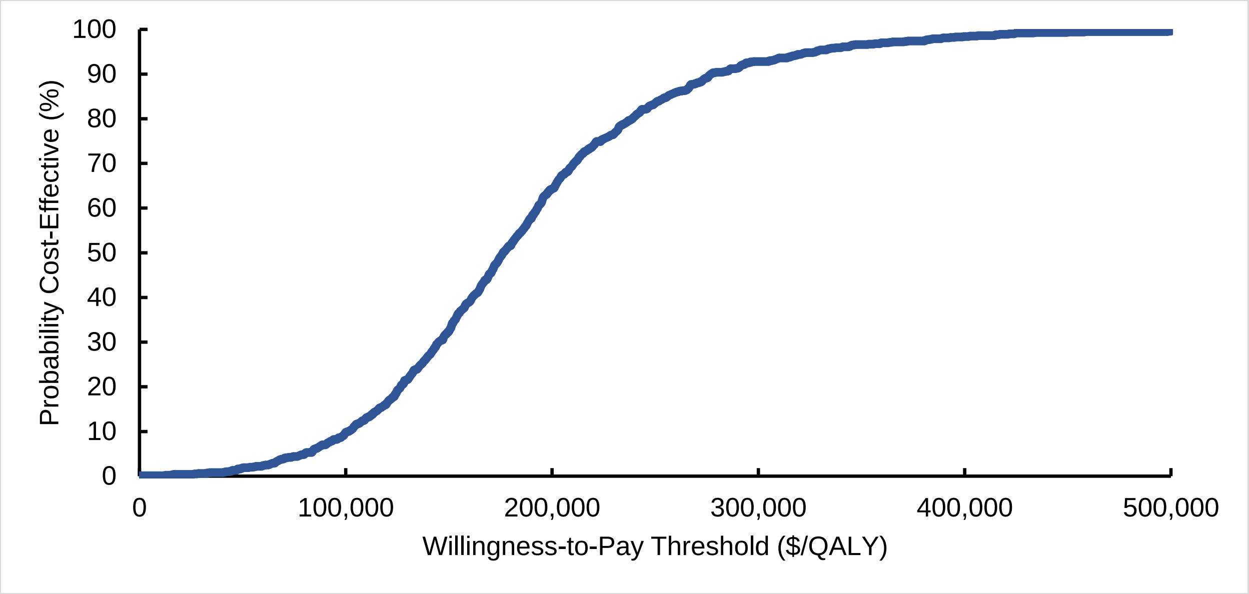

(b) Exploratory analysis.

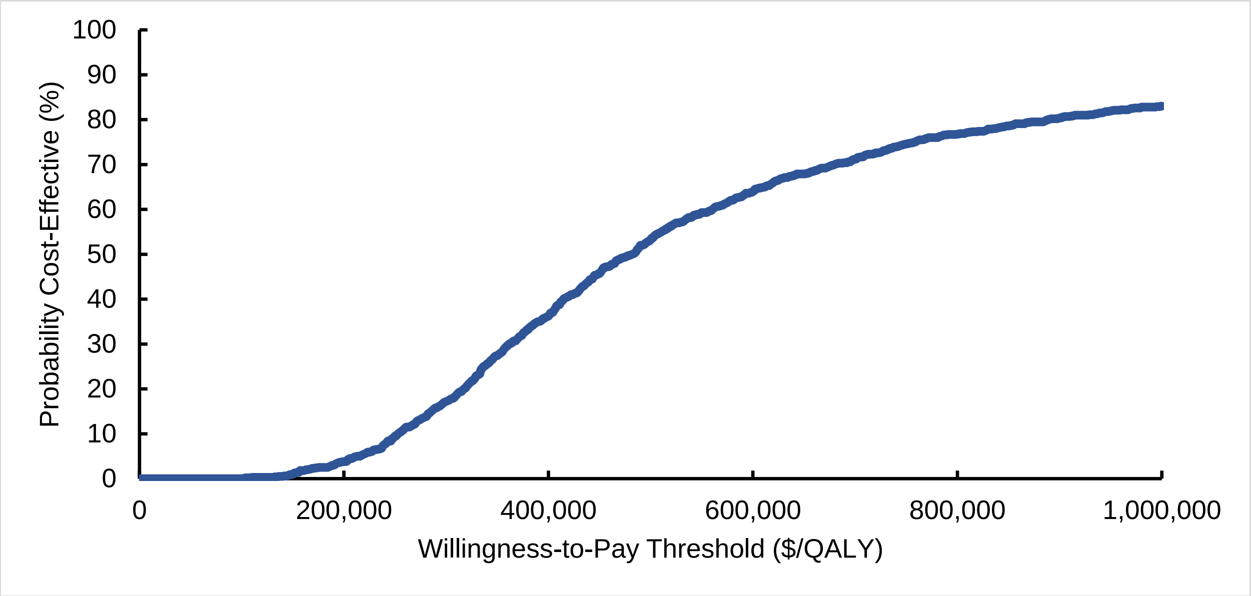

Abbreviations: QALY, quality-adjusted life-year

Each PSA was comprised of 1,000 iterations and sampled the distributions of 22 variables listed in eTable 2. We assumed triangular distributions for all variables except body weight, for which a normal distribution was used. The curve indicates the probability of pembro/axi being cost-effective as compared to nivo/ipi depending on the willingness-to-pay threshold. For the base case and exploratory analysis, pembro/axi had a 50% probability of being cost-effective at a willingness-to-pay threshold of approximately $176,000 and $482,000 per QALY, respectively.

© 2020 Watson TR et al. *JAMA Network Open.*

eMethods.

Cost Calculations

Costs for axitinib and cabozantinib were not provided in the ASP Drug Pricing Files. To determine the prices that Medicare would cover (model input), we adjusted the costs provided for axitinib and cabozantinib in the Micromedex Red Book. We determined the difference between the Red Book Average Wholesale Price (AWP) and ASP cost for pembrolizumab, nivolumab, and ipilimumab and lowered the Red Book AWP costs of axitinib and cabozantinib by the same proportion, 0.86, which was constant among the three drugs. The price of one cabozantinib tablet was constant regardless of the milligram dosage.[5,6]

On the basis of expert clinical opinion, we assumed that all patients who receive second-line therapy receive cabozantinib. Dosage and treatment duration were modeled after Choueiri, et al., 2015.[7]

BSC replaced general treatment costs in the last four months of life, on the basis of Henk, et al.'s analysis of BSC costs in metastatic RCC over a median time of 3.67 months. Henk, et al. did not specify the proportion of BSC costs that are paid by the patient out-of-pocket. We therefore used the patient liability percentage provided in Sheehan, et al.'s analysis and deducted this percentage from Henk's estimate to calculate the model input for BSC costs.[8,9]

Utility Calculations

Quality of life data from KEYNOTE-426 were not yet published at the time of our analysis. A utility value of 0.73 for metastatic RCC patients on first-line sunitinib was, however, available from a previous economic analysis supported by Pfizer, the manufacturer of sunitinib.[10] The utility calculation was based on data from the EuroQoL (EQ-5D) instrument in the phase 3 clinical trial leading to the approval of first-line sunitinib for metastatic RCC.[11] We judged that pembro/axi would be associated with a higher quality of life than sunitinib, on the basis of objective response rate (59.3% v. 35.7%) despite comparable toxicity as reported in KEYNOTE-426. We also assumed that quality of life on pembro/axi would be lower than that on nivo/ipi, on the basis of drug toxicity as reported in the clinical trials (rate of treatment-related grade three or higher adverse events, 62.9% v. 47%).[1,3] We therefore determined that an appropriate pembro/axi utility value would fall in between those for sunitinib and nivo/ipi.

© 2020 Watson TR et al. *JAMA Network Open.*

eReferences.

1. Rini BI, Plimack ER, Stus V, et al. Pembrolizumab plus Axitinib versus Sunitinib for Advanced Renal-Cell Carcinoma. *N Engl J Med.* 2019;380(12):1116-1127.
2. Motzer RJ, Tannir NM, McDermott DF, et al. Nivolumab plus Ipilimumab versus Sunitinib in Advanced Renal-Cell Carcinoma. *N Engl J Med.* 2018;378(14):1277-1290.
3. Motzer RJ, Rini BI, McDermott DF, et al. Nivolumab plus ipilimumab versus sunitinib in first-line treatment for advanced renal cell carcinoma: extended follow-up of efficacy and safety results from a randomised, controlled, phase 3 trial. *Lancet Oncol.* 2019;20(10):1370-1385.
4. Golshayan AR, George S, Heng DY, et al. Metastatic sarcomatoid renal cell carcinoma treated with vascular endothelial growth factor-targeted therapy. *J Clin Oncol.* 2009;27(2):235-241.
5. Centers for Medicare and Medicaid Services. October 2019 ASP drug pricing files. https://www.cms.gov/Medicare/Medicare-Fee-for-Service-Part-B-Drugs/McrPartBDrugAvgSalesPrice/2019ASPFiles. Accessed January 2, 2020.
6. IBM Micromedex RED BOOK: a comprehensive, consistent drug pricing resource. http://truvenhealth.com/Products/Micromedex/Product-Suites/Clinical-Knowledge/RED-BOOK. Accessed January 2, 2020.
7. Choueiri TK, Escudier B, Powles T, et al. Cabozantinib versus Everolimus in Advanced Renal-Cell Carcinoma. *N Engl J Med.* 2015;373(19):1814-1823.
8. Henk HJ, Chen C, Benedict A, Sullivan J, Teitelbaum A. Retrospective claims analysis of best supportive care costs and survival in a US metastatic renal cell population. *Clinicoecon Outcomes Res.* 2013;5:347-354.

© 2020 Watson TR et al. *JAMA Network Open.*

9. Sheehan DF, Criss SD, Chen Y, et al. Lung cancer costs by treatment strategy and phase of care among patients enrolled in Medicare. *Cancer Med.* 2019;8(1):94-103.

10. Remak E, Charbonneau C, Negrier S, Kim ST, Motzer RJ. Economic evaluation of sunitinib malate for the first-line treatment of metastatic renal cell carcinoma. *J Clin Oncol.* 2008;26(24):3995-4000.

11. Motzer RJ, Hutson TE, Tomczak P, et al. Sunitinib versus interferon alfa in metastatic renal-cell carcinoma. *N Engl J Med.* 2007;356(2):115-124.

© 2020 Watson TR et al. *JAMA Network Open.*
